# COVID-19 one year later: a retrospect of CRISPR-Cas system in combating COVID-19

**DOI:** 10.7150/ijbs.60655

**Published:** 2021-05-13

**Authors:** Yan Zhan, Xiang-Ping Li, Ji-Ye Yin

**Affiliations:** 1 Department of Clinical Pharmacology, Xiangya Hospital, Central South University, Changsha 410078, P. R. China; Institute of Clinical Pharmacology, Central South University; Hunan Key Laboratory of Pharmacogenetics, Changsha 410078, P. R. China.; 2 Engineering Research Center of Applied Technology of Pharmacogenomics, Ministry of Education, 110 Xiangya Road, Changsha 410078, P. R. China.; 3 National Clinical Research Center for Geriatric Disorders, 87 Xiangya Road, Changsha 410008, Hunan, P.R. China.; 4 Hunan Key Laboratory of Precise Diagnosis and Treatment of Gastrointestinal Tumor, Changsha 410078, P. R. China.; 5 Department of Pharmacy, Xiangya Hospital, Central South University, Changsha 410008, P. R. China.

**Keywords:** COVID-19, CRISPR-Cas, gene editing, SARS-CoV-2, diagnosis, mechanism research, therapeutics

## Abstract

Coronavirus disease 2019 (COVID-19), an infectious disease caused by Severe acute respiratory syndrome coronavirus 2 (SARS-CoV-2), has posed a persistent global threat. The transmission of SARS-CoV-2 is wide and swift. Rapid detection of the viral RNA and effective therapy are imperative to prevent the worldwide spread of the new infectious disease. Clustered Regularly-Interspaced Short Palindromic Repeats (CRISPR)- CRISPR-associated protein (Cas) system is an RNA-directed adaptive immune system, and it has been transformed into a gene editing tool. Applications of CRISPR-Cas system involves in many fields, such as human gene therapy, drug discovery and disease diagnosis. Under the background of COVID-19 pandemic, CRISPR-Cas system shows hidden capacity to fight the emergency in many aspects. This review will focus on the role of gene editing in COVID-19 diagnosis and treatment. We will describe the potential use of CRISPR-Cas-based system in combating COVID-19, from diagnosis to treatment. Furthermore, the limitation and perspectives of this novel technology are also evaluated.

## Introduction

Severe acute respiratory syndrome coronavirus 2 (SARS-CoV-2) is a coronavirus which caused an infectious disease named coronavirus infectious disease 2019 (COVID-19) 1. On March 11, 2020, the World Health Organization (WHO) announced that COVID-19 epidemic has constituted a pandemic. COVID-19 has posed a persistent global threat. As of March 2021, more than 100 million people were infected-confirmed and 2 million people died of this disease. Rapid detection of viral RNA and effective therapy are imperative to prevent the global spread of this new infectious diseases.

**Figure 1:**
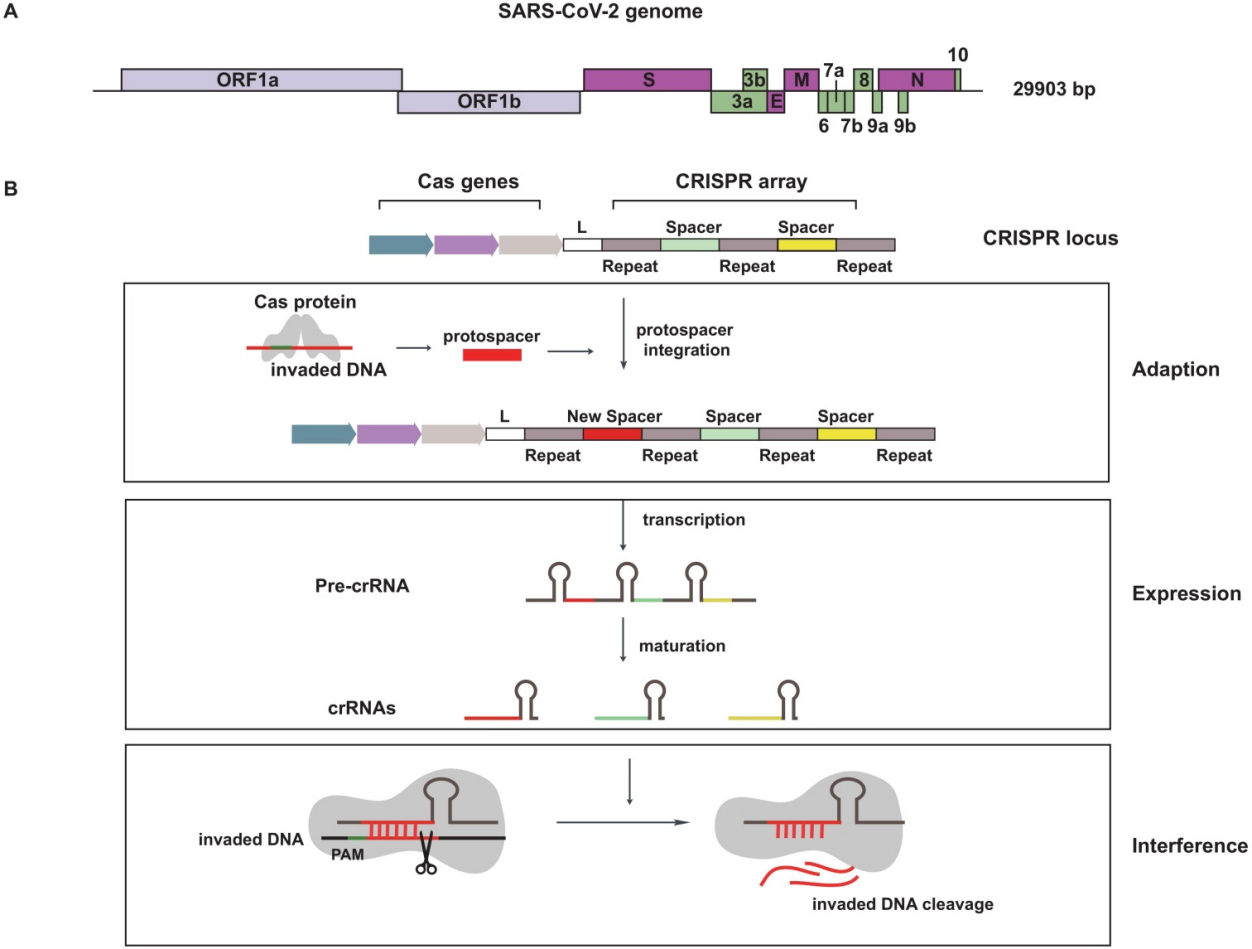
** Schematics for SARS-CoV-2 genome and the natural defense mechanism of CRISPR system.** (A) The skeleton diagram of SARS-CoV-2 genome. Light purple represents ORF1a and ORF1b, which encode non-structural proteins. Dark purple represents four structural protein-encoding genes. Green represents nine accessory protein-encoding genes. (B) A brief description of CRISPR-Cas system mechanism in defending foreign genetic material.

A basic understanding of SARS-CoV-2 is needed for fighting this virus. SARS-CoV-2 is an enveloped positive-sense RNA virus contained a single-stranded RNA genome of 29.9 kb (Figure 1A). The SARS-CoV-2 genome sequence is similar to SARS-CoV (~79%), but is distant from MERS-CoV (~50%). The genome is composed of 14 open reading frames (ORFs), most of them encode non-structural proteins (nsp1~nsp16) that participated in genome replication and RNA synthesis. The remaining parts encode nine accessory proteins (ORF3a~ORF10) and four structural proteins (spike, envelope, membrane and nucleocapsid), that are involved in the process of viral entry, assembly and release 2, 3.

The general detecting methods used for identifying SARS-CoV-2 are Metagenomic next-generation sequencing (mNGS) and Real Time Quantitative PCR (RT-qPCR) 4, 5. All methods that might work are recommended for treatment 6, 7. However, it is far from being enough to fight COVID-19 when people are confronted with huge infection burden. Any emerging technology that may be helpful is being valued. CRISPR-Cas system is being assessed. Researchers are making use of this technology in both therapeutic and molecular diagnostics of COVID-19 based on recent advances in this system. As a genome editing tool, CRISPR-Cas system can achieve precise editing, and simplify the experimental steps. It can provide rapid, inexpensive and highly sensitive diagnosis when used in nucleic acid detection. CRISPR is ahead of many new technologies for the features of high quality and cost-effective. We believe that it will play an important role in the fighting against the novel coronavirus.

## CRISPR-Cas system

CRISPR is a special repeated DNA sequences family and it is cooperated with Cas to constitute the CRISPR-Cas system 8. The first appearance of this magic combination is in 1987, but it wasn't until 2005 when CRISPR started catching people's attention. The CRISPR-Cas system is an RNA-directed adaptive immune system, which existed in approximately 48% of bacteria and 95% of archaea. When the invasion of foreign hereditary material is happening, the natural defense mechanism is triggered 9, 10. The whole defensive process including three stages: adaption, expression and interference (Figure 1B). In the stage of adaption, short direct repeats separated by spacers are short variable DNA sequences to form CRISPR sequence, and foreign DNA called protospacer is cleaved and integrated into CRISPR array. Therefore, these incorporated fragments become new spacers. The second stage is expression, CRISPR array is transcribed as precursor CRISPR-derived RNA (pre-crRNA), then pre-crRNA is matured to yield CRISPR-derived RNA (crRNA). Interference is the third stage, crRNA recruits and guides Cas effectors to specific target for cleaving nucleic acid derived from invading virus 11.

CRISPR-Cas system is divided into two classes based on different organization of effectors. Class 1 CRISPR-Cas system uses a huge effector complex consisting of multiple Cas proteins to deal with foreign nucleic acid, whereas Class 2 CRISPR-Cas system uses a single-protein effector 12, 13. Based on the effector protein families, class1 system is split into type I, type III, and type IV and class 2 system contains type II, V and VI systems. Cas9, Cas 12 and Cas13 belongs to type II, V, and VI, respectively.

Due to the difficulty of heterologous expression of multiple cascade complexes, class 2 CRISPR-Cas system is more convenient and rapid in dealing with emergencies, such as COVID-19, compared with class 1 system 14. Class 2 system is used in many fields, for example, (1) genome editing, including gene knock-out or knock-in that can be realized by a type II or type V effector enzyme for determining critical factors and providing potential therapy for diseases 15-17; (2) Targeting ssDNA/RNA for detection and treatment of viral diseases using type V or type VI effector 18.

**Figure 2:**
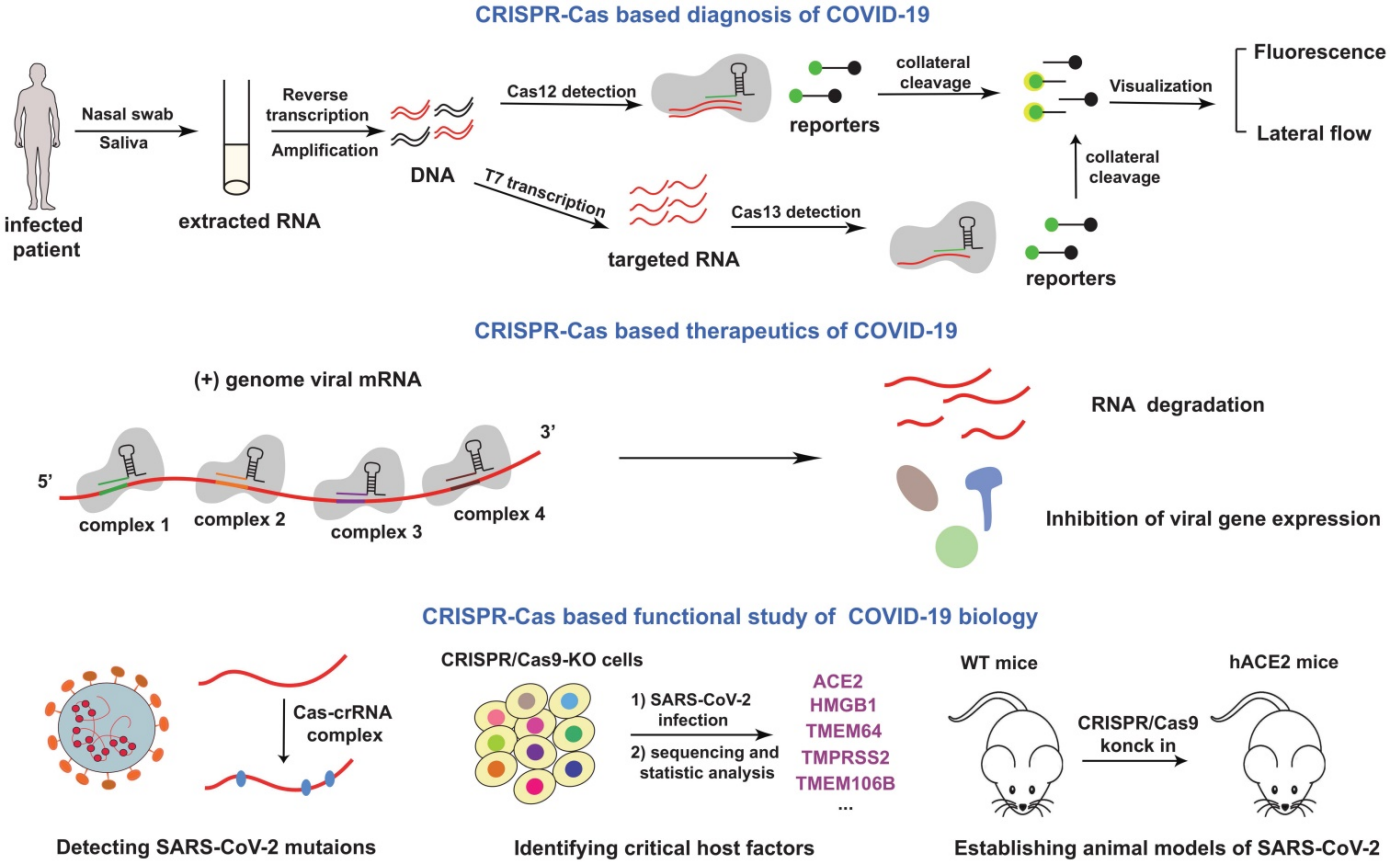
** Schematics for CRISPR-Cas based applications in COVID-19.** The mentioned contents were summarized in this figure. In the top of the picture, a cardinal principle of CRISPR-based diagnosis was shown. Researchers extracted patient sample RNA from nasopharyngeal or oropharyngeal swabs, and transformed RNA into cDNA with the process of reverse transcription. Then amplified virus cDNA, and use Cas protein targets predefined coronavirus sequences. The collateral cleavage of ssDNA probe confirmed detection of the virus. In the middle of the picture, the mechanism of therapeutics was depicted. Genome viral mRNA was degradation after CRISPR-Cas system cleavage, and the viral gene expression have also been inhibited. In the bottom of the picture, potential utilization of CRISPR in COVID-19 mechanism research was exhibited.

At present, the COVID-19 pandemic is still not under control. CRISPR-Cas system has the capacity to fight the disease. Although about 90% of all CRISPR-Cas loci identified in bacteria and archaea belong to the class 1 CRISPR- Cas system, most of the class 1 system are rarely used in eukaryotic genome engineering, because the common Cas proteins in class 1 CRISPR-Cas system have no specific functions. Based on these considerations, in our review, we mainly describe the related applications of class2 CRISPR-Cas system in combating the SARS-CoV-2 infection, which is the topic of general interest (Figure 2).

## CRISPR-Cas based diagnosis of COVID-19

COVID-19 is a public health emergency of international concern. Accurate and rapid diagnosis is essential for its early recognition and treatment. mNGS and RT-qPCR assays are the most commonly used technologies in detecting SARS-CoV-2. In the early days of the outbreak, mNGS is used to analyze the bronchoalveolar lavage fluid of patients and identified the virus 19. The advantages of mNGS are as follows: high throughput, unrestricted culture condition and comprehensive analysis. While its disadvantages are also obvious: high cost, complex operation process and long-time detection. In contrast, RT-qPCR reduces the analysis time, but it has high requirements for experimental operation. All problems mentioned above can be solved by CRISPR. CRISPR-Cas system can speed up the diagnostic process for its convenient operation and low cost. Advances in application of CRISPR-Cas system indicates its significance in fighting against COVID-19.

### CRISPR-Cas12 based diagnostic tools of COVID-19

CRISPR-Cas12 system is an extension of genomic editing tool. Cas12a, also called Cpf1, was identified first 20, then Cas12b (C2c1) and other Cas proteins in type V system were confirmed 21. The team of Jennifer Doudna found that type V CRISPR-associated proteins can stimulate non-specific single-stranded DNase activity to cut nearby dsDNA while Cas12 nucleases target dsDNA upon the mediation of guide RNA (gRNA). Hence, Jennifer et al. reported a method named DETECTER to realize quick and excellent nucleic acid detection for HPV-infected patient in clinics, which achieves attomolar sensitivity 22. The same principle is applied for the detection of SARS-CoV-2. Broughton et al. develops a CRISPR-Cas12-based assay for detection of SARS-CoV-2, which also called DETECTER. They extract patient sample RNA from nasopharyngeal or oropharyngeal swabs, and RNA is transcribed into cDNA with the process of reverse transcription. Using reverse tranion loop-mediated isothermal amplification (RT-LAMP) for isothermal amplification, the Cas12 targets predefined coronavirus sequences, the E and N genes of SARS-CoV-2, after which cleavage of ssDNA probe confirms detection of the virus. The system can be run in approximately 30-40 min and visualized on a lateral flow strip, which is faster and more specific than traditional RT-qPCR 23. SARS-CoV-2 DETECTER is a powerful instrument for COVID-19 diagnosis and scientists are contributed to optimizing the diagnostic procedures. Ma et al. described using the MeCas12a system to detect SARS-CoV-2 by targeting the E gene. They screened manganese ion to enhance the signal up to 13‐folds for the tested crRNAs, which enable the limit of detection to reach five copies and improve specificity. It also can distinguish infection of SARS‐CoV‐2 from MERS‐CoV in the simulated clinical samples 24. Another analogous platform is ENHANCE. Nguyen et al. developed the system with engineered crRNAs and optimized conditions that enabled to detect various clinically relevant nucleic acid targets, including human immunodeficiency virus, hepatitis virus C and SARS-CoV-2, with high sensitivity 25. Apart from the increasing sensitivity and specificity, researchers setup to simplifying the diagnostic procedures. Ding et al. presented an AIOD-CRISPR assay for simple, ultrasensitive, one-pot, and visual detection of SARS-CoV-2. In the AIOD-CRISPR assay, all components used for nucleic acid amplification and CRISPR-based detection are thoroughly mixed in a single-tank reaction system and incubated at one temperature, eliminating the need for separate pre-amplification and transfer of culture amplification products. Meanwhile, double crRNA without PAM sequence restriction is introduced to launch CRISPR-based SARS-CoV-2 detection 26. This procedure has fewer steps and dramatically reduces the chance of operation-introduced nucleic acid contamination or cross-contamination among samples. STOPCovid is described as a concise tool targeting N gene for detection, which combines simplified extraction of viral RNA with isothermal amplification and CRISPR-mediated detection. Considering loop-mediated isothermal amplification (LAMP) operates at 55 to 70 °C, researchers choose Cas12b as protein effector which is a thermostable Cas enzyme 27. This same conditions also apply in CASdetec, a system which acts on RNA-dependent RNA polymerase (RdRp) of SARS-CoV-2 28. In addition, there have some other optimizations, including introducing microfluidics technology into CRISPR-based diagnostics 29, 30, and adding the quenched fluorescent single-stranded DNA (ssDNA) reporter generating fluorescence signal visible to the naked eye 31, 32. The summary of Cas12-based SARS-CoV-2 diagnostics in presented in Table 1.

### CRISPR-Cas13 based diagnostic tools of COVID-19

Cas13a (C2c2) is an important part of CRISPR-Cas13 system (type VI) specifically targeting RNA 14, 33. Dr. Zhang and his colleagues confirmed that Cas13a is a single-component programmable RNA-guided RNA-targeting CRISPR effector and they employed Cas13a to develop new RNA-targeting tools 34. CRISPR-Cas13 based diagnostics can be used to detect invading RNA due to its collateral cleavage of nearby non-targeted RNAs 35. SHERLOCK was created for this reason. Gootenberg et al. use SHERLOCK to detect Zika and Dengue virus with high sensitivity and specificity 36, 37. SHERLOCK was also used to identify Ebola virus and Lassa virus 38. Since the outbreak of CODVID-19, people are eager looking for diagnosis to identify symptomatic, asymptomatic, and pre-symptomatic carriers of the virus. The team of Feng Zhang reformed SHERLOCK to make it feasible for SARS-CoV-2 detection. They redesigned different primers and guide-RNA for targeting open reading frame 1ab (ORF1ab) and spike (S) gene. Clinical validations suggest that test result of SHERLOCK is identical to RT-qPCR 39, 40. SHERLOCK is not a multi-purpose system. It is time-consuming when dealing with massively multiplexed nucleic acid detection. It also has a lot of hidden dangers. SHINE was created to seek the optimal conditions to realize recombinase polymerase amplification (RPA)-based amplification and Cas13-based detection occurring in one step, which simplifying the whole streamline and available for testing unextracted samples 41. CARMEN can comprehensive test large SARS-CoV-2 sample sets based on its intrinsic multiplexing and throughput capabilities 42. DISCoVER is a RNA extraction-free SARS-CoV-2 test which applies saliva-based sample matrix to detect virus 43. Careful crRNA selection, and proper combinations between crRNA and Cas13 will optimize SARS-CoV-2 detection. Introducing emerging technologies will avoid the need for amplification and reverse transcription. Fozouni et al. designed many crRNAs along the N gene of SARS-CoV-2 to determine the best, and replaced Cas13 with its homolog for highest sensitivity and robust *trans*-cleavage activity 44. Tian et al. applied microfluidic technology to CRISPR-Cas13-based system for nucleic acid amplification-free single-molecule RNA detection 45. There are other platforms show outstanding performance in SARS-CoV-2 detection. All Cas13-based SARS-CoV-2 diagnostics are comprehensively summarized in Table 2.

### CRISPR Cas9-based diagnostic tools of COVID-19

CRISPR-Cas9 is well-known for its strong gene-editing capacity 46. Moreover, Cas9 provides excellent DNA recognition capability without trans-cleavage activity, which is used for development of biosensors. In the COVID-19 diagnosis, scientists utilize dead Cas9 (dCas9), a mutation of Cas9, to realize the function of virus detection. dCas9 was generated by the simultaneous mutation of RuvC1 and HNH regions of cas9 endonuclease. The endonuclease activity of dCas9 disappeared completely, and only the ability of gRNA to guide into the genome was retained. Therefore, dCas9 can achieve multiple functions by binding to other effector proteins. Researchers reported a colorimetric viral detection method based on CRISPR/dCas9 system. The dcas9 gRNA complex was fixed on the well plate, and then viral lysate and biotin-PAMmer were added. After incubation and washing, streptavidin horseradish peroxidase (HRP) and 3,3′,5,5′-tetramethylbenzidine (TMB) reagent were added to the plate. Through the oxidation of TMB, yellow light could be observed for the viral RNA 47.

Xiong et al. realized the rapid and visual detection of two SARS-CoV-2 genes (ORF1ab and E) in a single test and a single strip at the same time based on CRISPR/Cas9‐Mediated Lateral Flow Assay 48. There are other detection platforms based on CRISPR-Cas9 49, 50, such as CASLFA, which has realized accurate diagnosis of Listeria monocytogenes, genetically modified organisms, and African swine fever virus. The application of Cas9-based SARS-CoV-2 diagnostics are presented in Table 2.

## CRISPR-Cas based functional study of COVID-19 biology

At present, the functional study of COVID-19 biology mainly considered three aspects: virus, host and their interaction *in vivo*. We need understand these parts in order to better conquer the virus. CRISPR-Cas system has many contributions in these fields.

### Detecting mutations of SARS-CoV-2

Coronavirus is prone to mutation in the process of evolution because of its particular genome structure. Thousands of SARS-CoV-2 genome sequences reveal a number of mutations 51. D614G is a missense mutation which leads to an amino acid change from aspartate to a glycine residue at position 614 in the S protein 52. The mutation was first discovered in Europe. After several months of transmission, it has become the main genotype. The ability of SARS-CoV-2 to invade human is enhanced due to D614G mutation. Thus powerful methods for its fast and reliable detection in very important 53. Wang et al. proposed a method based on a CRISPR-Cas13 amplification principle to profile mutated variants of SARS-CoV-2 54. At the same time, Meng et al. used engineered Cas12a gRNA to detect the SARS-CoV-2 D614G mutation 55.

### Screening critical host factors for SARS-CoV-2 infection

The first step of the virus attacking human is infection. The spike protein of the virus binds to the receptor located in host cells, and then release a small single stranded RNA into it, which can be used as a messenger RNA to cheat the ribosome and synthesize RNA replicase. RNA replicase will generate RNA negative strand according to the viral RNA, and this RNA negative strand will continue to generate more RNA fragments and RNA positive strand. These different RNA fragments and ribosomes generate more different viral protein structures. Finally, complete viral particles are formed, that can be excluded from cells and infect new cells. Therefore, it is very important to find out the key host factors affecting viral infection. Using genome-wide screening based on CRISPR gene editing technology, scientists identified several potential host factors related to the viral invasion. Daniloski et al. found that knockout of a set of genes (*ATP6AP1*, *ATP6V1A*, *NPC1*, *RAB7A*, *CCDC22*, and *PIK3C3*) induces transcriptional changes in cholesterol biosynthesis pathway and reduces viral entry 56. Wei et al. revealed that* HMGB1* regulates *ACE2* expression and is critical for the entry of SARS-CoV-2 57. *TMEM106*, *BVMP1*, *TMEM41*, and *TMEM64 (VTT)* domain-containing protein *transmembrane protein 41B (TMEM41B)* were significant for infection by SARS-CoV-2 58, 59. *ACE2* and *TMPRSS2* were also screened out and ranked as the top genes 60. Baggen et al. discovered that lysosomal protein TMEM106B is important for SARS-CoV-2 to infect human cell lines and primary lung cells 61. Not only were they important in virus infection, but host factors screened by CRSISPR-Cas system also can be used to predict adverse drug response or improve drug efficacy. Akinci et al. found that loss of *SLC29A3* mitigates remdesivir toxicity but not change SARS-CoV-2 antiviral potency, and that the mitochondrial adenylate kinase *AK2* is a kinase required for the remdesivir efficacy and toxicity 62. These screened host factors represent a rich resource to develop new therapeutic strategies for combating COVID-19.

### Establishing animal models of SARS-CoV-2

Studies in SARS-CoV-2 should not stay at the cellular level, but also need appropriate animal models to seek some clues for combating COVID-19. Human angiotensin-converting enzyme II (*ACE2*) is a functional receptor for SARS-CoV-2 entry. Researchers use CRISPR-Cas9 knock-in technology to generate a mouse model expressing human ACE2 (*hACE2*). The transgenic mouse would be a useful tool for studying SARS-CoV-2 transmission and pathogenesis and testing potential vaccines and therapeutics 63-65.

## CRISPR-Cas based therapeutics for COVID-19

It is known that CRISPR-Cas13 is an RNA-guided RNA-targeting CRISPR system. The characteristic of this system may be used to destroy the RNA of SARS-CoV-2 and inhibit its replication capacity. Nguyen et al. used CRISPR-Cas13d system to disrupt the virus. CRISPR-Cas13d system consists of two parts, Cas13d protein and gRNAs which contained spacer sequences specifically complement with the virus RNA genome. They designed 10,333 guide-RNA for targeting 10 peptide-coding regions of SARS-CoV-2, and treat adeno-associated virus as a vector to deliver the Cas13d effector 66. PAC-MAN is created based on the same operating principle. To find a best region for specific targeting viral RNA, Abbott et al. utilized bioinformatic analysis, a group of six crRNAs that can target 91% of sequenced coronaviruses and a group of 22 crRNAs enabled to target all sequenced coronaviruses were revealed finally. PAC-MAN becomes a potential antiviral strategy 67. The latest study published in February 2021 displayed that CRISPR-Cas13a system can mitigate SARS-CoV-2 infection, and this conclusion is identified in hamsters 68. In a word, Cas13 can be used *in vivo* against SARS-CoV-2.

## Limitation and future perspective

Diagnostics based on CRISPR-Cas system is a rapid and simple method. At the beginning of using CRISPR system in nucleic acid detection, it requires a large quantity of targeted RNA molecules. In addition, the false positive rate was a concern. With continuous improvement of this technology, CRISPR-Cas-based diagnosis achieves attomolar sensitivity. Compared with RT-qPCR, it avoids aerosol pollution, which reduces false positive rate. mNGS can relieve the disadvantage of RT-qPCR, and improve the positive detection of CRISPR system. However, the analysis time of mNGS is too long, and the results cannot be produced in time. CRISPR system has many advantages.

Mechanistic researches based on CRISPR-Cas system is expanding. Recently, a new viral strain, B1.1.7, was found in UK. The virus strain had 23 mutations, N501Y is a concerning one 69. It changes the most important part of the spike protein, the receptor binding domain that the virus uses to contact with human respiratory cells. Currently, conventional methods are still used for viral detection. Although there is no relevant literature about CRISPR system applied to N501Y detection, CRISPR will make contributions for its detection.

Therapeutics based on CRISPR-Cas system provide a potential option for treating COVID-19. The risk of off-target cannot be ignored in treatment. There are two solutions for off-target effect, optimize design of sgRNA and improve the specificity of Cas protein. Scientists have come up with a lot of possible measures and the risk of off-target effect has been low thus far. Another limitation is applicability of CRISPR-Cas system. CRISPR is not widely used in human. Most of the current research has been carried out in animals, and this limitation should be overcome one day.

In conclusion, in the context of COVID-19 pandemic, novel technologies will help advance the disease diagnosis and therapeutics.

## Figures and Tables

**Table 1:** CRISPR-Cas12 based COVID-19 detection assays

Platform Name	Cas protein	Time	Sensitivity	Specificity	Visualization	Target genes	Reference
contamination-free visual detection	Cas12a	40 min	100%	100%	Lateral flow	ORF1ab, N, E	69
SENA	Cas12a	N/A	99%	99%	Fluorescence	ORF1ab, N	70
opvCRISPR	Cas12a	45 min	N/A	N/A	Naked eye	S	31
iSCAN	Cas12a	40 min	86%	100%	Fluorescence/lateral flow	N, E	71
ITP-CRISPR	Cas12a	30-40 min	93.8%	100%	Fluorescence	N, E	29
CRISPR-FDS	Cas12a	15 min	100%	100%	Fluorescence	ORF1ab	30
CRISPR/Cas12a-NER	Cas12a	45 min	N/A	N/A	Fluorescence	E	32
STOPCovid.v1	Cas12b	50 min	N/A	N/A	Lateral flow	N	27
STOPCovid.v2	Cas12b	15-45 min	93.1%	98.5%	Fluorescence/lateral flow	N	27
DETECTR	Cas12a	45 min	95%	100%	Lateral flow	E, N	23
MeCas12a	Cas12a	45 min	100%	100%	Naked eye	E	24
ENHANCE	Cas12a	40-60 min	N/A	N/A	Fluorescence/lateral flow	N	25
AIOD-CRISPR	Cas12a	20 min	N/A	N/A	Naked eye	N	26
COVID-19 CRISPR-FDS	Cas12a	50 min	100%	71.4%	Fluorescence	ORF1ab, N	72
CRISPR-ABC	Cas12a	30-40 min	91.2%	99.2%	Fluorescence	ORF1ab	73
OR‑DETECTR	Cas12a	50 min	N/A	N/A	Fluorescence	RdRp, N	74
CASdetec	Cas12b	60 min	N/A	N/A	Naked eye	RdRp	28

**Table 2:** CRISPR-Cas13/Cas9 based COVID-19 detection assays

Platform Name	Cas protein	Time	Sensitivity	Specificity	Visualization	Target genes	Reference
SHERLOCK	Cas13a	40 min	100%	100%	Fluorescence	ORF1ab, N, S	39
SHINE	Cas13a	50 min	90%	100%	Smartphone/lateral flow	ORF1ab	41
DISCoVER	Cas13a	30 min	N/A	100%	Fluorescence	N	43
CARMEN	Cas13a	N/A	N/A	N/A	Fluorescence	N/A	42
Ultralocalized Cas13a assay	Cas13a	N/A	single-molecule	single-nucleotide	Fluorescence	ORF1a, N	45
TL-LFA	Cas9	< 1 h	100%	97.1%	Fluorescence	ORF1ab, E	48

## Abbreviations

SARS-CoV-2: Severe acute respiratory syndrome coronavirus 2
COVID-19: coronavirus disease 2019
ORFs: open reading frames
RT-qPCR: Real Time Quantitative PCR
CRISPR: Clustered Regularly-Interspaced Short Palindromic Repeats
Cas: CRISPR-associated protein
pre-crRNA: precursor CRISPR-derived RNA
crRNA: CRISPR-derived RNA
dsDNA: double-stranded DNA
RT-LAMP: reverse tranion loop-mediated isothermal amplification
LAMP: loop-mediated isothermal amplification
PAM: protospacer adjacent motif
RdRp: RNA-dependent RNA polymerase
ssDNA: single-stranded DNA
ORF1ab: open reading frame 1ab
S: spike
N: nucleocapsid
E: envelope
M: membrane
RPA: recombinase polymerase amplification
gRNA: guide RNA
dCas9: dead CRISPR-associated protein 9
PAMmer: adjacent motif -presenting oligonucleotide
HRP: horseradish peroxidase
TMB: 3,3′,5,5′-tetramethylbenzidine
hACE2: human angiotensin-converting enzyme II
